# Systematic Review and Meta-Analysis of Work-Related Musculoskeletal Disorder Prevalence Among European Surgeons: Effect of Demographic, Economic, and Quality-of-Life Indicators

**DOI:** 10.3390/ijerph23030398

**Published:** 2026-03-21

**Authors:** Philippe Gorce, Julien Jacquier-Bret

**Affiliations:** 1 Université de Toulon, CS 60584, CEDEX 9, 83041 Toulon, France; gorce@univ-tln.fr; 2 International Institute of Biomechanics and Occupational Ergonomics, Avenue du Docteur Marcel Armanet, CS 10121, 83418 Hyères, France

**Keywords:** musculoskeletal disorders, SBS ratio, HDI, body area, surgeons, healthcare professionals, quality of life, meta-analysis, systematic review, Europe

## Abstract

**Highlights:**

**Public health relevance—How does this work relate to a public health issue?**

- WMSDs are ubiquitous among healthcare professionals.
- Knowledge of prevalence and the factors that cause them is essential for good health at work and maintaining the effectiveness of interventions by surgeons.

**Public health significance—Why is this work of significance to public health?**

- To have data on the general prevalence and by body area among surgeons in Europe for the development of new health policies (recommendations and education).
- This study investigates new parameters such as HDI and SBS as risk factors underlying WMSD.

**Public health implications—What are the key implications or messages for practitioners, policy makers and/or researchers in public health?**

- For practitioners: to carry out their activities in the best working conditions while protecting their health.
- Improved quality of life for surgeons.

**Abstract:**

Background: Work-related musculoskeletal disorders (WMSDs) are common among surgeons. The objective was to study how economic, demographic, and quality-of-life indicators influence the WMSD prevalence among European surgeons. Methods: A systematic review and meta-analysis was conducted according to the Preferred Reporting Items for Systematic Reviews and Meta-Analyses guidelines. Three open databases were scanned without a date limit to extract the overall WMSD prevalence and by body area. Results: Among the 11,814 articles, 25 were included, with a total of 5174 surgeons. The overall prevalence was pooled at 75.8% (95% CI: 68.6–83.1%). The most affected areas were lower back (52.1%, 95% CI: 43.1–61.0%), neck (51.2%, 95% CI: 43.7–58.9%), shoulder (43.1%, 95% CI: 36.4–49.8%) and upper back (34.2%, 95% CI: 24.2–44.1%). Surgeons aged over 45 with more than 10 years’ experience had a higher prevalence of WMSDs in the neck, hip, upper and lower back. The overall and lower back prevalence was higher among surgeons in countries where the surgery-by-surgeon ratio was greater than 125. A negative correlation was observed between the Human Development Index and the lower back prevalence. Conclusions: Further research is needed to strengthen ergonomics programs, knowledge, and organizational work strategies to effectively reduce WMSD prevalence among European surgeons.

## 1. Introduction

A surgeon’s work activity is important for the health of a country’s population. It is characterized by devotion in part to consultations; to administrative tasks; and lastly, to performing surgery related to their specialty. Surgical procedures require a high level of concentration and precision [1,2]. In addition, surgery can place a significant cognitive load on the surgeon, which can be exacerbated by the surgical environment, the operating room team, and the complexity of the intervention [3]. The physical load is also significant, particularly due to awkward static postures maintained for a long time [4]. All of these working conditions expose surgeons to work-related musculoskeletal disorders (WMSDs). These manifest as inflammatory and degenerative conditions of the bone, muscle, nerve, or ligament tissues [5]. In the long term, these disorders lead to the development of pathologies in different body areas (e.g., rotator cuff disease, tenosynovitis, osteoarthritis, sciatica, carpal tunnel syndrome, low back pain, etc.) [6,7,8].

Assessing the WMSD prevalence is a hotspot among surgeons as the number of studies dedicated to them is very high [9,10]. The results showed that, as with all healthcare professionals [11,12,13], the prevalence of WMSDs among surgeons exceeds 80% [14,15] and has been pooled at 68.1% (95% CI: 63.5–72.7%) based on 69 worldwide studies [16]. The most affected areas were the lower back, neck, shoulder, and wrist. Several surveys reported a prevalence of WMSDs for the lower back [14,17,18] and neck [19,20] of around 60% and a prevalence of around 50% for the shoulder and wrist [21,22].

However, as highlighted by worldwide meta-analyses [23,24], considerable heterogeneity can be observed in overall prevalence and prevalence by body region. This variability can be explained by numerous methodological, demographic, psychological, sociological, geographical, or environmental factors. A commonly used solution to reduce this heterogeneity is to conduct subgroup analyses to study the effect of one or more parameters on the prevalence of WMSDs. For demographic characteristics, authors have shown an effect of age [25], experience [26], or BMI [27]. Other authors have studied the effect of working conditions, such as the number of hours worked per week [28], overtime or night shifts [29], or time spent standing [27], etc. Other more macroscopic parameters may also impact the prevalence of WMSDs. Healthcare spending determines the level of care provided to the population and the working conditions of healthcare professionals, particularly surgeons. This spending is generally expressed as a percentage of Gross Domestic Product (GDP). In Europe, these expenditures average 10% of countries’ GDP [30]. Investments can help improve these working conditions, particularly by upgrading hospital infrastructure, increasing the number of healthcare personnel, and improving the quality of ergonomic equipment in order to reduce the risk of WMSDs. The Human Development Index (HDI) is another macroscopic parameter that could have an effect on the WMSD prevalence. It is a national indicator that links the population’s level of education to its life expectancy [31]. The application of ergonomic knowledge can lead to improvements in pain levels, physical workload, and quality of life among surgeons [32]. Physical workload is an important environmental factor in the development of WMSDs that must be taken into consideration. For surgeons, it is often represented by the number of cases treated or the number of surgeries performed over a given period of time [33]. Studies have shown that increased time spent performing surgery leads to an increase in the prevalence of WMSDs [27]. However, this information is not always reported, and there is no macroscopic quantitative indicator to represent the phenomenon. For nurses, for example, workload is characterized by the nurse-to-bed ratio, which links the number of active nurses to the number of patients to be treated at the department, facility, or national level. In particular, it has been shown that a higher nurse-to-bed ratio reduces the prevalence of WMSDs [34,35]. A similar index could provide further insights into the prevalence of WMSDs among surgeons. Finally, recent analyses by continent are also a means of reducing heterogeneity and highlighting geographical factors linked to the incidence of WMSDs [16,36]. To our knowledge, no meta-analysis has been conducted in European countries to investigate the links between economic and geographical factors and the prevalence of WMSDs.

Thus, the objective was to produce a quantitative summary of the overall WMSD prevalence and their injuries for all areas of the body among surgeons in Europe, regardless of their specialty or department. The literature was scanned to compile a list of relevant works and provide a systemic overview supplemented with a meta-analysis. Subgroup analyses based on demographic, economic, and quality-of-life parameters were conducted to further evaluate WMSDs. One of the original aspects of this work was the introduction of a new national indicator representing the average number of surgeries performed per surgeon at the country level, called the surgery-by-surgeon ratio. The value of this analysis lies in the fact that it provides reliable data for the development of specific interventions that can be generalized to health policies aimed at reducing the risk of MSDs among surgeons.

## 2. Materials and Methods

### 2.1. Search Strategy

The search was conducted between 16 and 31 October 2025, using the following combination of keywords linked by the Boolean operators AND and OR without publication date restriction: (“work-related musculoskeletal disorders” OR WMSD*) AND surgeon AND prevalence. Three free databases were explored: Google Scholar, PubMed/Medline, and ScienceDirect without additional artificial intelligence assistance.

The authors’ names, year of publication, title, journal, and database of origin for each record were manually compiled into a single Excel spreadsheet. Excel’s duplicate removal function was used on the titles to eliminate multiple entries. From the list of unique items, two reviewers (P.G. and J.J.-B.) assessed each article based on its title and abstract to retain only cross-sectional studies that investigated the prevalence of WMSDs among European surgeons (application of inclusion/exclusion criteria). The two lists compiled by the two reviewers were compared. Any differences were discussed and resolved by consensus to establish the list of articles to be evaluated in detail. Then, the full texts were retrieved and assessed separately by the two reviewers to retain only those that met all the inclusion/exclusion criteria. The final list was obtained by comparing the lists of articles selected by the two reviewers. Any discrepancies were resolved through re-evaluation and consensus. In cases where it was not possible to reach a decision, the opinion of a third reviewer was solicited.

### 2.2. Inclusion and Exclusion Criteria

Population, Exposure, Comparator, and Outcomes (PECO) guidelines were applied to establish the inclusion criteria [37]. An article was included if it was a cross-sectional study reporting the prevalence of WMSDs (O) among European surgeons (P) in their professional practice, regardless of their specialty. The main outcome (O) was the overall prevalence and prevalence by body area. The choice of body areas was based on those generally used in the international literature. These are the neck, upper back, lower back, shoulders, elbows, wrists, hips, knees, and ankles. The definition proposed by Kuorinka et al. in the Nordic Musculoskeletal disorders Questionnaire (NMQ [38]) was used to define WMSDs: “symptoms such as pain and discomfort lasting at least one week or occurring at least once a month during the last 12 months.” When a study used the NMQ, it was included. When it did not use the NMQ, it was included if the prevalence assessment was conducted by body area over a 12-month period. Comparisons (C) are not applicable in this study.

Five exclusion criteria were considered: (1) the study design was other than a cross-sectional study; (2) the study was not written in English; (3) it was not original peer-reviewed research (book, chapter, report, case report, case study, conference, thesis, etc. were excluded); (4) the population includes subjects from various healthcare professions with no way to separate prevalence rates by profession; (5) insufficient details on prevalence by body area.

### 2.3. Quality and Risk of Bias Assessment

Cross-sectional studies were assessed using the AXIS tool [39]. The 20 items of the AXIS tool were grouped into five domains: (1) objectives and design (items 1 to 4), (2) population selection (items 5 to 9), (3) variable measurement (items 10 to 13), (4) statistical analyses (items 14 to 17), and (5) generalization and transparency (items 18 to 20). The overall judgment of the risk of bias was based primarily on the items in domains 2 to 4, considered critical for WMSD prevalence studies. The risk of bias classification for each domain was as follows: low risk if all critical items had a “YES” response or if at most one critical criterion was defined as “No” or “Unclear”; moderate risk if 2 to 3 critical items had a “No” or “Unclear” response; high risk if more than 4 items had a “No” or “Unclear” response. The overall risk of bias in the study was assessed on a three-level scale: low, moderate, or high. A low risk was determined when all three critical domains received a low risk rating and at most one non-critical domain received a moderate risk rating. A high risk was assigned if at least two critical domains received a high-risk rating. Otherwise, the overall risk was defined as moderate.

For each included study, two reviewers independently evaluated the 20 items on the AXIS grid and the overall risk based on the five domains assessment. Any discrepancies were discussed to achieve the final assessment. The results are presented in a traffic-light plot [40].

### 2.4. Data Extraction

Two reviewers independently extracted the following information from each included article: name of the first author, year of publication, country, sample size, type of questionnaire, male/female ratio, mean age, BMI, number of years of experience as a surgeon, specialty, hours worked per week and average duration of surgical procedures (in cases or hours per week), overall prevalence of WMSDs and prevalence of WMSDs for each reported body area. It was found that prevalence was not always reported for the overall sample. When it was reported for a subgroup of subjects, prevalence was reported for the total sample in order to obtain consistent data and ensure comparability between studies. The extractions made by the two evaluators were compared and any discrepancies were resolved by consensus. The extracted data were summarized in two separate tables, with empty cells indicating missing information.

For each European country involved in the selected studies, the population, GDP, HDI, number of surgical procedures per 100,000 inhabitants, and number of surgeons per 100,000 inhabitants were collected [41,42,43,44] from global statistical sources. Based on the number of surgeons and the number of surgical procedures per 100,000 inhabitants, the Surgery-By-Surgeon (SBS) ratio was computed:

$$SBS\;ratio=\frac{Surgery\;per\;100,000\;Inhabitants}{Surgeons\;per\;100,000\;Inhabitants}$$

### 2.5. Certainty Assessment

The quality of evidence in this meta-analysis was assessed using GRADE (Grade of Recommendations Assessment, Development and Evaluation). The two reviewers (P.G. and J.J.-B.) separately rated the five GRADE domains, namely risk of bias, indirectness of evidence, inconsistency of results, imprecision of results, and publication bias, and then assigned the overall GRADE rating on a scale of four levels: high, moderate, low, and very low. The two evaluations were then compared and discussed to validate the final rating.

### 2.6. Statistical Analysis

The overall prevalence of WMSDs in Europe and the prevalence by body area were pooled from all studies without any transformation or variance correction using the method described by Neyeloff et al. [45]. The pooled prevalence for each area was reported with its normal 95% confidence interval (95% CI). For each meta-analysis, heterogeneity among studies was measured using Cochran’s Q test (significance level < 10%) and the I^2^ statistic. The level of heterogeneity was defined by four levels as suggested in the Cochrane Training [46]: low (I^2^ < 40%), moderate (I^2^ between 30% and 60%), substantial (I^2^ between 50% and 90%), and high (I^2^ > 75%). The application of a fixed-effect or random-effects model was determined by combining the two parameters I^2^ and p(Q): for p(Q) ≥ 0.1 and I^2^ ≤ 50%, a fixed-effects model was selected for the meta-analysis (no heterogeneity found between studies). Conversely, a random-effects model was applied if p(Q) < 0.1 and I^2^ > 50% (significant heterogeneity observed). According to Neyeloff’s spreadsheet, no random effects estimator or proportion transformation was used.

A workload (case load per week), two demographic (age, years of experience) and three country indicators (HDI, GDP, SBS ratio) were considered to perform subgroup analyses and investigate their effect on overall and body area WMSD prevalence among European surgeons. According to the literature, a division into two subgroups was conducted for age (under 45 years, over 45 years [47]), experience (under 10 years, over 10 years [48]), and SBS ratio (under 125, over 125, choice based on Gadjradj et al. [47] observations, with 66% of surgeons performing between 0 and 300 surgeries per year). For GDP, HDI, and case load per week, a meta-regression was performed. Finally, the effect of the year of publication on prevalence was studied. Meta-regressions were achieved using JASP (v0.19.3.0). The significance threshold was set at 5%. For studies including subjects from several countries without possible distinction, subgroup analyses cannot be conducted for the three country indicators (HDI, GDP, SBS ratio).

### 2.7. Registration and Report

The Preferred Reporting Items for Systematic reviews and Meta-Analyses (PRISMA) guidelines [49,50] were followed to perform the systematic review and report the results (see Supplementary Material). The protocol was registered in the PROSPERO database (CRD420251185499).

## 3. Results

### 3.1. Search Results

**Figure 1:**
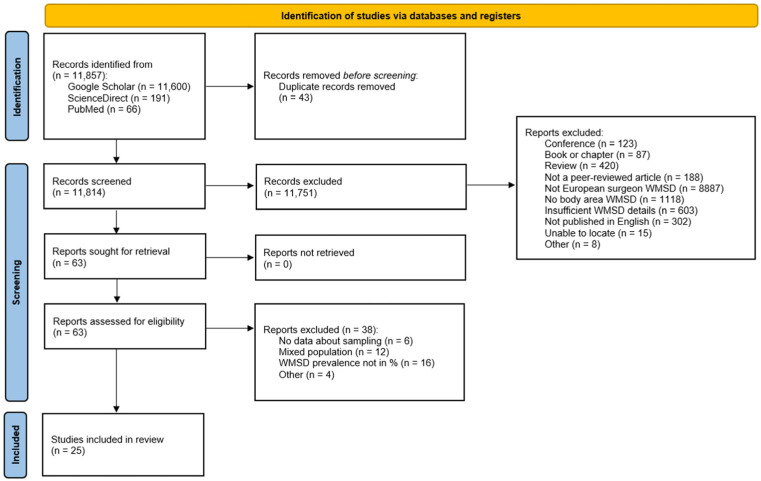
PRISMA flow diagram.

A total of 11,857 records were identified from the search of the three databases. Forty-three duplicates were removed using the Excel function. Among the 11,814 unique items, 11,751 were excluded based on the inclusion/exclusion criteria. The majority (8887 studies) were excluded because the study had not been conducted in a European country. The others were excluded because they were not original peer-reviewed studies or because they did not provide sufficient information on the WMSD prevalence. Sixty-three articles were evaluated based on their full text, and 38 were excluded due to a mixed sample that did not allow data to be extracted for surgeons or a prevalence that could not be used for meta-analysis. Ultimately, 25 cross-sectional studies were included in the present systematic review for a total of 5174 surgeons. The full process is presented in Figure 1.

### 3.2. Study Characteristics

Table 1 presents the demographic data for each study included. First, the study conducted by Stomberg et al. [51] investigated the prevalence of WMSDs for two surgical specialties, for which all reported information was clearly separated. We therefore considered these two populations as two separate studies. The total number thus increased to 26 studies (Table 1 therefore has 26 rows). Nine European countries are covered: Germany (2 studies [20,52]), Ireland (2 studies [53,54]), Italy (3 studies [33,55,56]), The Netherlands (3 studies [57,58,59]), Romania (1 study [18]), Spain (3 studies [22,60,61]), Sweden (2 studies in one [51]), Turkey (1 study [62]), and United Kingdom (7 studies [7,26,63,64,65,66,67]). Two studies were conducted in several European countries (marked as “Multiple” in Table 1 [68,69]). The sample sizes varied greatly, ranging from 17 [56] to 751 [52]. Twelve specialties are represented in the various studies: cardiology (8 surgeons), gastroenterology (88 surgeons), general surgery (200 surgeons), gynecology (290 surgeons), neurosurgery (21 surgeons), ophthalmology (518 surgeons), orthopedics (12 surgeons), otorhinolaryngology (2554 surgeons), plastic surgery (14 surgeons), thoracic surgery (8 surgeons), urology (293 surgeons), and vascular surgery (10 surgeons). Nine studies included a total of 1158 surgeons from different specialties without detailing the composition of the sample (marked as “Multiple” in Table 1). The surgeons who participated in the studies were predominantly male (65.2% vs. 34.8% across 18 studies). Only three studies had a predominantly female population [51,52,54]. The average age was 46.3 ± 0.5 years based on 15 studies, and the average experience was 17.1 ± 1.0 years computed from the 12 available records. Other demographic data were rarely reported in most studies. Only three studies reported an average BMI [18,52,61] and two studies reported the average number of hours worked per week [18,52]. Workload was reported in two different ways: either by the number of cases per week (3 studies [18,22,33]) or by the number of hours per week (10 studies).

Table 2 presents the data for each country that will be used in the subgroup analyses. The GPD of European countries ranges from €353.8 billion (Romania) to €4305.6 billion (Germany), and their HDI ranges from 0.845 (Romania) to 0.972 (Ireland). The SBS ratio has been computed between 42.4 (Ireland) and 339.8 (The Netherlands). No data was available for Romania.

Table 3 depicts the WMSD prevalence reported in each study. Five studies reported prevalence for all nine body regions, two of which also reported overall prevalence (reported in 21 studies in total). The most studied area was the neck (23 studies), followed by the shoulder (21 studies), lower back (20 studies), and wrist (18 studies). Prevalence was assessed much less frequently for the lower limbs: hip (7 studies), ankle (8 studies), and knee (12 studies). Prevalence was studied with a Nordic Musculoskeletal Disorders Questionnaire [38] in five studies. The other studies used a questionnaire specifically developed for their analysis (Table 1).

### 3.3. Quality Appraisal and Risk of Bias

**Figure 2:**
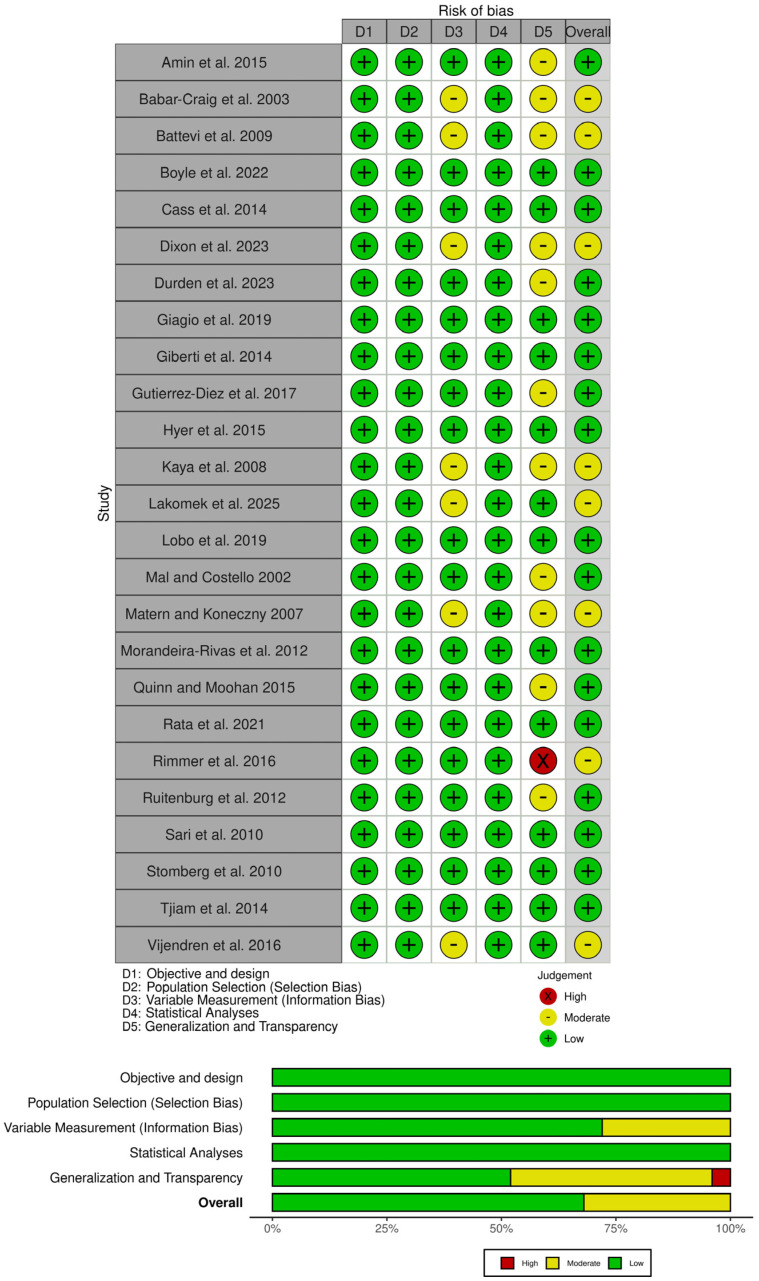
Representation of risk of bias of each included study. Reference: [7,18,20,22,26,33,51,52,53,54,55,56,57,58,59,60,61,62,63,64,65,66,67,68,69].

Figure 2 presents the overall assessment of the risk of bias for each study based on the five domains of AXIS. Eight studies were evaluated with a moderate risk of bias [20,52,55,62,64,67,68,69]). The remaining 17 studies presented a low risk of bias [7,18,22,26,33,51,53,54,56,57,58,59,60,61,63,65,66]. The detailed quality assessment with AXIS tool was presented in Appendix A.

### 3.4. Meta-Analysis of WMSD Prevalence

**Figure 3:**
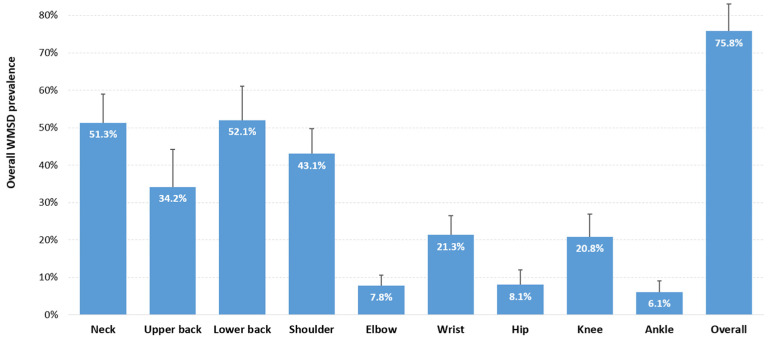
WMSD prevalence (overall and by body area) pooled from data available in each included study. The vertical bars represent the 95% confidence intervals.

Figure 3 shows the WMSD prevalence among European surgeons, pooled from the values reported in each included study. The overall prevalence was estimated at 75.8% (95% CI: 68.6–83.1%, 21 studies, 4505 surgeons). As illustrated, the two most affected areas were the lower back (52.1%, 95% CI: 43.1–61.0%, 29 studies, 3996 surgeons) and the neck (51.2%, 95% CI: 43.7–58.9%, 32 studies, 4797 surgeons) with a prevalence higher than 50%, followed by the shoulder (43.1%, 95% CI: 36.4–49.8%, 30 studies, 4004 surgeons) and upper back (34.2%, 95% CI: 24.2–44.1%, 22 studies, 2440 surgeons). A prevalence of 20% was observed for the wrist (21.3%, 95% CI: 16.1–26.5%, 27 studies, 2729 surgeons) and the knee (20.8%, 95% CI: 14.7–27.0%, 21 studies, 2038 surgeons). With a prevalence less than or equal to 8%, the elbow, hip, and ankle were the least exposed body areas.

### 3.5. Subgroup Meta-Analyses

Table 4 illustrates the effect of age on overall prevalence and prevalence by body area. Surgeons over 45 years had a higher WMSD prevalence for neck (53.0% vs. 46.0%) and hip (20.4% vs. 9.3%), while the wrist prevalence decreased compared to the younger group (17.6% vs. 24.6%). Prevalence rates were similar between the two groups for other areas, including overall prevalence.

Table 5 details the effect of experience as a surgeon on the prevalence of WMSDs. First, the results showed a higher overall prevalence of WMSDs for surgeons with more than 10 years of experience (74.7% vs. 66.4%). This increase was observed for the neck (56.8% vs. 32.9%), upper back (44.7% vs. 23.5%), and lower back (58.3% vs. 25.9%), for which rates above 40% and even 50% were measured for the most experienced group. On the other hand, a decrease in prevalence with years of practice was observed for the wrist (18.4% vs. 24.0%) and knee (23.0% vs. 29.1%). The effect of experience could not be tested for the hip and ankle due to a lack of data in the group with less than 10 years of experience.

The effect of the SBS ratio computed for each country is shown in Table 6. A 10% higher overall WMSD prevalence was found for surgeons with a ratio greater than 125 (81.1% vs. 71.4%), as well as for the lower back (51.4% vs. 40.4%). There were no variations in other areas except for the wrist, where the prevalence decreased as the ratio increased (19.0% to 26.6%).

**Figure 4:**
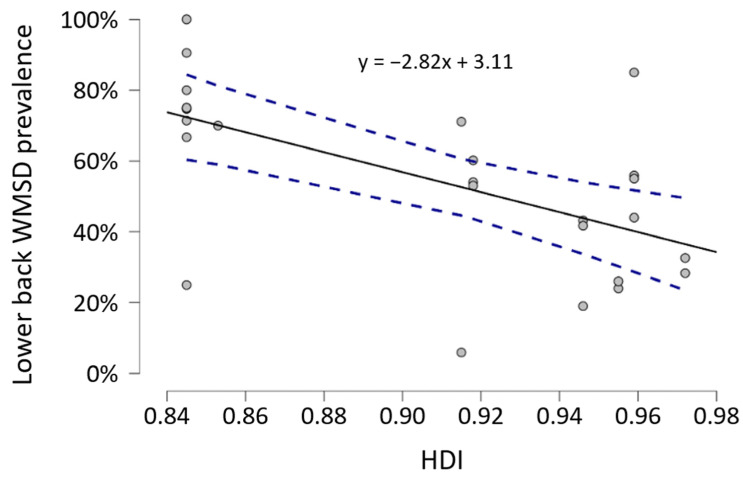
Meta-regression illustrating the relationships between HDI and lower back WMSD prevalence (including 27 data). Each circle represents a study and the dotted lines represent the 95% confidence interval.

Table 7 summarizes the results of meta-regressions performed to study the effect of GDP, HDI, and case load (hour per week) on the WMSD prevalence. Only the lower back prevalence was significantly correlated with HDI: an increase in HDI could lead to a reduction in the lower back prevalence with a Pearson’s r coefficient of −0.603 (*p* < 0.05, 20 studies, Figure 4). Another correlation was observed entre the year of publication and the overall and upper back WMSD prevalence (r = 0.469, 21 studies and r = 0.624, 13 studies respectively, *p* < 0.05).

### 3.6. Publication Bias Tests

**Figure 5:**
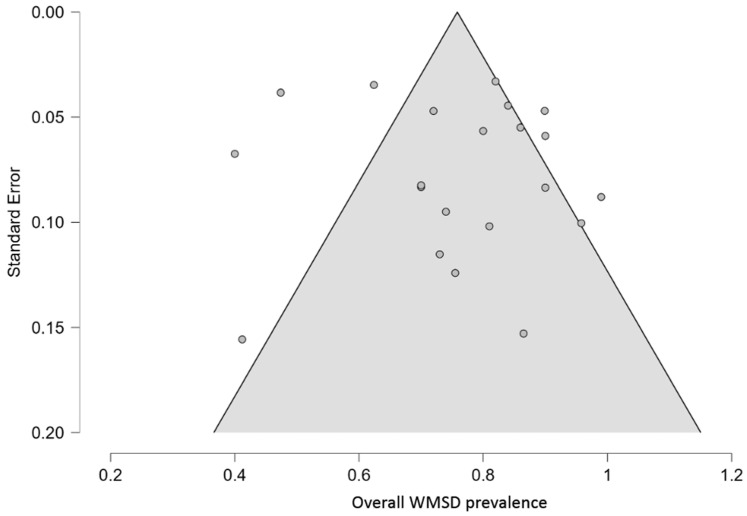
Funnel plot to test the publication bias of the studies included in the meta-analysis.

The funnel plot indicated that there was no significant publication bias for overall WMSD prevalence (Figure 5). The results of Eggers’s test also did not reveal statistically significant publication bias for neck, lower back, and shoulder. However, the analysis revealed asymmetry for the other six body areas. This result suggests a potential risk of publication bias, likely due to the possible presence of small-study effects. Nevertheless, this interpretation should be considered with caution given the limited number of studies for some areas and the observed heterogeneity.

### 3.7. Sensitivity Analysis

**Figure 6:**
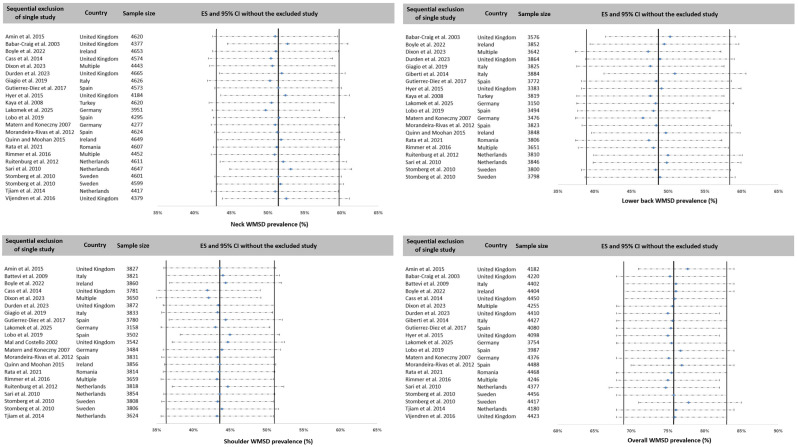
Sensibility analysis by successive one by one exclusion for neck, lower back, shoulder and overall WMSD prevalence. Reference: [7,18,20,22,26,33,51,52,53,54,55,56,57,58,59,60,61,62,63,64,65,66,67,68,69].

Two sensitivity analyses were conducted. The first was performed on the overall prevalence and on the three most affected areas, i.e., lower back, neck, and shoulder, by successively excluding studies one by one (Figure 6). The mean variations were less than 1% compared to the pooled prevalence across all studies. The highest difference was 1.7% for neck and shoulder, 2.0% for overall prevalence, and 2.2% for lower back. Because the data collection tool was likely to affect the prevalence reported by the studies, the second sensitivity analysis consisted in comparing the prevalence obtained by separately grouping studies that used a standardized questionnaire (NMQ) and those that used a questionnaire specifically developed for their study. With the exception of the upper back, which showed a 15.5% difference between the two groups, the difference was less than 5% for all areas and 6% for overall prevalence (Table 8). These small differences highlighted the good stability of all the results presented.

### 3.8. Level of Evidence

There is a fair degree of certainty that surgeons’ practices are associated with variations in the risk of WMSDs. However, the results of our meta-analysis indicate a very low overall quality of evidence for all body regions. As only cross-sectional studies were included, the initial level of evidence according to the GRADE scale was low due to the observational methodology. The heterogeneity observed for all areas was high (I^2^ > 50%) and the 95% confidence intervals were wide (95% confidence interval difference > 50% of the event rate) for half of them. Egger’s tests did not reveal publication bias only for neck, lower back, shoulder, and overall WMSD prevalence. For these reasons, the level of evidence was downgraded for all areas. The detailed assessment of certainty and GRADE level of evidence is available in Appendix B.

## 4. Discussion

The aim of this study was to conduct a systematic review and meta-analysis to investigate the WMSD prevalence (overall and across 9 body areas) among European surgeons. Twenty-five cross-sectional studies were considered for a total of 5174 surgeons. A subgroup analysis was performed to assess the effect of demographic (age, experience and BMI), economic (GDP), and quality of life (HDI) parameters on WMSD prevalence. An original national parameter was introduced, namely the SBS ratio (average number of surgeries per surgeon), in order to quantify its effect on this prevalence.

### 4.1. WMSD Prevalence

The overall prevalence ranged from 40% to 99% with a pooled mean of 75.8% (95% CI: 68.6–83.1%, 4505 surgeons) from the 21 studies that reported this data. This prevalence appears to be higher than the value observed in international meta-analyses conducted by Gorce et al. (68.1%, 68 studies, 17,188 surgeons [16]) or Daruwalla et al. [70] (71.1%, 56 articles, 13,628 surgeons). This prevalence is also slightly higher than data reported in a continental study [16], i.e., 73.1% for the European subgroup included 16 studies. Recent studies included in the present work showed that the WMSD prevalence is increasing despite numerous awareness and training campaigns that should have helped to reduce the very high risk [71]. There are several possible explanations. On the one hand, recent technological advances have made it possible to perform video- and robot-assisted surgery that are more ergonomically favorable and potentially less stressful than conventional surgery, in addition to being beneficial for the patient [72]. However, it has been shown that the use of these new technologies exposes surgeons to a greater WMSD risk [72,73,74]. This aspect is all the more relevant given that 50% of the sample in the present study consisted of otorhinolaryngology practitioners who mainly operate with equipment. As observed in several included studies, the overall prevalence is greater than 80% [52,61] for this specialty, which contributes to a slightly overestimated the overall WMSD prevalence among surgeons. Working conditions could play a role in this increase. Although precautions are taken to make the workspace ergonomic (equipment, posture, etc.), the distribution of the workload (caseload per week or SBS ratio) can take over and counteract the beneficial effects of these adjustments. In fact, it is common for surgeons to operate during specific periods (e.g., a half-day or a full day) rather than spreading their procedures throughout the week for material and organizational reasons. Thus, the significant repetition or maintenance of awkward static postures, particularly standing, for very long periods of time, combined with a heavy mental load, can have negative consequences despite an adapted environment. Dianat et al. [27] reported an increased risk of WMSDs when the time spent operating exceeded 25 h per week, when standing work exceeded 4 h per day, and when a surgery lasted more than 3 h. The areas most affected by surgery were the lower back and neck, with more than one in two surgeons (52.1% and 51.2% respectively) affected, followed by the shoulder and upper back, with one in three surgeons (43.1% and 34.2% respectively) affected. This result is consistent with the majority of studies conducted worldwide [70,75], including analyses by continent [16]. The neck and upper back follow the same trend as the overall prevalence: despite health policies aimed at reducing WMSDs, the prevalence of these two areas continues to increase (Table 8). It is therefore necessary to continue and find solutions to protect surgeons in their practice in order to stem this increase. The risk is that, in the long term, there will be a shortage of surgeons, mainly due to the specific nature of the practice and the physical and mental strain that negatively impacts their personal and professional quality of life [71].

### 4.2. Demographic Parameters and WMSD Prevalence

An age effect on the prevalence was observed. The results showed a higher prevalence among surgeons aged over 45 for the neck and hip. Other studies have reported that age is a risk factor for the development of WMSDs [21,76]. Al-Mohrej et al. [77] showed a higher prevalence for hip, knee, and ankle among surgeons over 32 years old. The lower limbs are heavily stressed by maintaining static standing postures for long periods during surgery, which increases the risk of WMSDs [27]. With advancing age, a significant deterioration in neuromuscular function is observed. Strength and velocity parameters decrease with age, particularly isometric strength [78]. This could therefore explain the higher prevalence of WMSDs in the lower limbs in subjects older than 45. On the other hand, the prevalence in the wrist decreases with age. This result was also observed by Rata et al. [18]. However, this result contrasts with those proposed by Al-Mohrej et al. [77], who observed an increase with age. One explanation could be related to the nature of the samples. In the study by Rata et al. [18], prevalence was pooled without taking into account the surgeons’ specialty, as in the present analysis, whereas in the study by Al-Mohrej et al. [77], the effect was reported for orthopedic surgeons. It therefore appears that specialty has a direct effect on the prevalence of WMSDs. The demands, levels of precision, and duration of surgery can vary significantly between specialties and are therefore parameters that can influence the prevalence assessment. One solution would be to conduct subgroup analyses by specialty to refine the effect of age on the prevalence of WMSDs.

An effect of experience was observed on the overall prevalence. Surgeons with more than 10 years of experience were more exposed (74.7% vs. 66.4%). This result has been observed in other studies in the literature. Gadjradj et al. [47] reported that surgeons with less than 15 years of experience were four times less likely to be affected by WMSDs. Michael et al. [79] found a significant difference in overall prevalence of more than 50% between a group of novice surgeons and a group with 15 to 20 years of experience. The authors who conducted the analyses by body area highlighted an increase in prevalence for the wrist, hip, knee [77], and leg [80]. These results contradict those obtained in the present analysis. One explanation could be the heterogeneous sample coupled with a small number of studies in which prevalence was measured in surgeons with less than 10 years of experience. In fact, for these four areas, the number of studies was less than or equal to 2, i.e., too limited to perform a meta-analysis. A similar conclusion can be drawn for the increase observed for the neck and back (lower and upper). It seems difficult to draw further conclusions without a significant increase in the number of studies, which would increase the robustness of the results obtained.

### 4.3. GDP, HDI and WMSD Prevalence

A subgroup analysis was performed to study the link between GDP of each country and WMSD prevalence. Despite the significant difference in GDP between European countries, i.e., with a maximum ratio of 12 between Romania (€353.8 billion) and Germany (€4305.3 billion), no significant correlation was observed for overall prevalence or prevalence by body area. Few studies have addressed this macroscopic correlation. To our knowledge, only Jacquier-Bret et al. [81] have studied this relationship in nurses. The authors found a positive correlation between GDP and the prevalence of hip and knee pain on the African continent. These results are quite surprising. One might have thought that a higher level of resources would be associated with a reduction in prevalence due to investments made, in particular, in a more ergonomic working environment and greater material or organizational resources. Therefore, the redistribution of resources alone does not ensure the protection of surgeons. One reason could be related to the inclusion of both public and private practices in the sample. Although no effect was identified for caseload in our study, it has been shown that in the private sector, the number of interventions could be up to twice as high [82]. A subgroup analysis considering both the resources invested in the field of health dedicated to the prevention of WMSDs, rather than GDP, which is too macroscopic, and the separation between public and private sectors could provide a better understanding of their effects on the prevalence of WMSDs among surgeons. Furthermore, GDP is a parameter that reflects the resources allocated at the national level, whereas in most studies, prevalence rates are often reported at the level of a department or institution, i.e., at the local level. It is highly likely that the resources of some departments or institutions do not reflect the value of GDP (much more or much less resources), which can significantly affect the measurement of WMSD prevalence. Consequently, the relationship between resource levels and prevalence needs to be studied in greater depth, particularly by considering the correlation between an institution’s level of development (human, financial resources, structural, number of beds, etc.) and the associated level of WMSD risk.

The HDI is also a macroscopic indicator that, in addition to economic factors, includes life expectancy and access to education. The European countries represented in this study all have an HDI above 0.8, indicating that they belong to the very high human development tier [43]. This reflects widespread access to affordable education and healthcare, characteristics of countries with stable politics and dynamic economies where inhabitants enjoy high life expectancy and quality of life. Our results showed that even among countries with a very high HDI, a higher value was correlated with a reduction in WMSD for the lower back. This area has often been identified in the literature as being the most exposed among surgeons [83,84]. A longer life experience necessarily implies a longer working time. As has been shown in the literature, a positive effect of age on the prevalence of WMSD has been demonstrated [21,77]. Therefore, a reduction in prevalence with an increase in HDI would be linked to the level of knowledge of practitioners in the country in question. Several studies conducted among healthcare professionals have shown that this knowledge had a direct effect on the WMSD presence. Hess et al. [71] proposed an ergonomic program as a potential solution for effectively reducing risk factors in order to optimize the professional performance of surgeons in the operating room. Külekçioğlu et al. [32] showed that surgeons who had undergone ergonomic training and exercise programs experienced significant improvements in pain, physical workload, and quality of life. However, surgeons still lack knowledge of ergonomics [85]. It therefore seems important to continue and strengthen awareness and training policies in the context of WMSD prevention.

### 4.4. SBS Ratio and WMSD Prevalence

The SBS ratio is the relationship between the number of surgeries performed and the number of practicing surgeons in a given country over one year. Like the HDI, this dimensionless ratio enables the level of surgical activity to be quantified and countries to be compared with one another. In this study, this new indicator showed a large difference between European countries, despite the fact that they are all developed countries with a very high HDI. Indeed, the country with the highest SBS ratio has a value of 339.8 (The Netherlands), compared to only 42.4 in Ireland. Subgroup analysis showed that surgeons with an SBS greater than 125 (i.e., 1 surgery every 3 days, 2.4 per week, or 10.4 per month) had a higher overall WMSD prevalence (81.1% vs. 71.4%) and lower back WMSD prevalence (51.4% vs. 40.4%) (Table 7). With SBS ratio, a higher surgical workload is a risk factor for WMSD in surgeons. The advantage of this parameter is that it provides standardized information at the country level. This result could not be replicated with caseload (number of surgeries per week or per month), another parameter sometimes used in the literature [86,87]. Indeed, this parameter is not always reported, which was the case in our systematic review, as it was only mentioned in three of the cross-sectional studies included. When this information is reported, it is very often well above the threshold of 125 surgeries per year [88,89]. Under these conditions, the question of how this workload is distributed appears to be important (should these procedures be performed over several days, several half-days, or one day?), as does the type of procedure (assisted or unassisted [23]) and the duration of the procedures [90]. Comparing the prevalence observed for these different modalities could provide a better understanding of the causes of WMSDs and thus enable recommendations to be made for prevention.

The results also showed a reduction in prevalence at the wrist with increasing SBS ratio, age, and experience. Thus, experienced surgeons (older and more experienced) have greater control over their practice at the upper extremities despite the precision and concentration required. However, this result was obtained by pooling all specialties. Some authors have highlighted that specific specialists had a higher prevalence at the wrist [91]. A subgroup analysis by specialty among European surgeons would identify the specialties for which the wrist is less exposed.

### 4.5. Limitations

Several limitations should be considered. The first concerns data heterogeneity. Significant heterogeneity was observed across studies for the majority of analyses. Despite subgroup and sensitivity analyses, and the use of a random-effects model, heterogeneity remained high. This suggests potential differences in methodological approaches and population characteristics across studies. Indeed, significant differences were observed in demographic parameters (age, sex, height, weight, experience, etc.), sample sizes, types of procedures (open or minimally invasive surgery, with or without assistance), and specialties. Furthermore, it is important to note that otolaryngologists are significantly overrepresented compared to other specialties. This overrepresentation limits the generalizability of the results to the entire profession. Subgroup analyses generally reduce this variability, but it remained significant in our results, mainly because the number of studies was still too small relative to the number of subgroups. This heterogeneity, combined with the observational design of the study, creates a potential risk of bias that is important to consider. Consequently, pooled estimates should be interpreted with caution and require further investigation. From a statistical perspective, applying transformation methods (e.g., logit) would help mitigate the effects of a non-normal prevalence distribution or asymmetric confidence intervals, particularly for prevalence close to 0 or 1. From a methodological point of view, it is important to identify the major factors that have a significant effect on the estimation of prevalence. These factors—whether methodological, related to anthropometric data, demographic factors, or working conditions—can then be used and combined to conduct subgroup analyses and better manage this heterogeneity. Given this multifactorial nature, this relies on cross-sectional studies that report prevalence data by subgroup according to these various criteria within a defined and common methodological framework. An increase in the number of such studies is also essential for generalizing the results and understanding the mechanisms underlying the onset of WMSDs, which are undeniably present among surgeons.

The second limitation relates to the method used to collect WMSD prevalence data. The use of non-standardized questionnaires in the majority of studies leads to considerable variability in the reporting of results. One way to compensate for this variability is to consider only studies conducted using a standardized questionnaire such as the NMQ [92]. In addition, the subjective nature of the responses is also a source of variability that must be taken into account. All these factors have led to a loss of information, particularly on demographic parameters. As a result, some subgroup analyses could not be performed due to the absence of these data.

Three of the parameters used for subgroup analyses are defined at the country level. However, some cross-sectional studies were conducted at different sites in several countries, which meant that they could not be used in these analyses, thereby reducing the amount of data available.

The decision to prioritize original peer-reviewed research articles published in English may have led to the exclusion of relevant work, which is another limitation.

## 5. Conclusions

European surgeons are exposed to WMSDs, primarily affecting the lower back, neck, and shoulder. Subgroup analyses have shown that demographic factors (age and experience) increase WMSD prevalence. The introduction of the SBS ratio revealed that WMSD prevalence increases beyond 125 surgical procedures. Future work could incorporate more parameters to reduce data variability. Further research is needed to strengthen ergonomics programs, knowledge, and organizational work strategies to effectively reduce WMSD prevalence.

## Figures and Tables

**Table 1:** Detailed characteristics of the 26 included studies.

Authors	Country	Sample Size	Questionnaire	Specialty	Male/Female (%)	Age * (Year)	BMI	Experience * (Year)	Working Hour (h per Week)	Case Load * (Case per Week)	Case Load * (h per Week)
Amin et al., 2015 [63]	United Kingdom	82	Self	Otorhinolaryngology		47.9		15.04			
Babar-Craig et al., 2003 [64]	United Kingdom	325	Self	Otorhinolaryngology							
Battevi et al., 2009 [55]	Italy	88	Self	Gastroenterologist	68.2/31.8	42.8		15.1			
Boyle et al., 2022 [53]	Ireland	49	Self	Otorhinolaryngology	73.5/26.5						
Cass et al., 2014 [26]	United Kingdom	128	Self	Gynecologic							
Dixon et al., 2023 [68]	Multiple (Europe)	259	Self	Multiple							
Durden et al., 2023 [65]	United Kingdom	37	NMQ	Multiple		48					
Giagio et al., 2019 [33]	Italy	76	NMQ	Multiple	65.8/34.2	37.7 ± 12.1		10.4 ± 11.3		16.1	18.9
Giberti et al., 2014 [56]	Italy	17	NMQ	Multiple	94.1/5.9	51.3		3			6.0
Gutierrez-Diez et al., 2017 [60]	Spain	129	NMQ	Multiple	61.2/38.8	42.0 ± 12.3		16 ± 12			17.0 ± 9.7
Hyer et al., 2015 [66]	United Kingdom	518	Self	Ophthalmologists	74.5/25.5	48.7					8.78
Kaya et al., 2008 [62]	Turkey	82	Self	Multiple							
Lakomek et al., 2025 [52]	Germany	751	Self	Otorhinolaryngology	47.3/52.7	51 ± 11	24.0 ± 5.0	23.87 ± 11.0	40.33 ± 12.0		4.08 ± 7.0
Lobo et al., 2019 [61]	Spain	407	Self	Otorhinolaryngology	51.5/48.5	44.9 ± 11.4	24.55 ± 3.2	17.1 ± 11.1			
Mal and Costello, 2002 [7]	United Kingdom	367	Self	Otorhinolaryngology		51.2					
Matern and Koneczny, 2007 [20]	Germany	425	Self	Multiple	75.0/25.0						
Morandeira-Rivas et al., 2012 [22]	Spain	78	Self	Multiple	87.0/13.0	44.06				1.3	
Quinn and Moohan, 2015 [54]	Ireland	53	Self	Gynecologic	36.0/64.0	30.4		4.5			
Rata et al., 2021 [18]	Romania	95	NMQ	Multiple	62.1/37.9	37.56 ± 8.74	25.44 ± 5.04	10.09 ± 8.41	41.03 ± 14.62	7.56 ± 3.73	13.07 ± 7.27
Rimmer et al., 2016 [69]	Multiple (Europe)	250	Self	Otorhinolaryngology	78.8/21.2						
Ruitenburg et al., 2012 [57]	The Netherlands	91	Self	General	55.0/45.0	41.0 ± 10.8					
Sari et al., 2010 [58]	The Netherlands	55	Self	Multiple	65.4/34.6	39.5 ± 7.3		8.75 ± 6.07			6
Stomberg et al., 2010 [51]	Sweden	101	Self	Gynecologic	36.6/63.4	48.2 ± 10.2		14.5 ± 10.4			1.9
Stomberg et al., 2010 [51]	Sweden	103	Self	General	82.2/17.8	43.3 ± 9.1		8.7 ± 5.1			3.0
Tjiam et al., 2014 [59]	The Netherlands	285	Self	Urologic	93.0/7.0	46		12.9			6.45
Vijendren et al., 2016 [67]	United Kingdom	323	Self	Otorhinolaryngology				18.7			

* Age, year of experience, and case load are presented as mean (±standard deviation when available). BMI: Body Mass Index; NMQ: Nordic Musculoskeletal Questionnaire.

**Table 2:** Description of parameters by country included in the analyze.

Country	Country Population (Million)	GDP * (Billion €)	HDI	Surgery per 100,000 Inhabitants	Surgeons per 100,000 Inhabitants	SBS Ratio
Germany	84.1	4305.3	0.959	19,124	108.0	177.1
Ireland	5.3	562.8	0.972	3053	72.0	42.4
Italy	59.2	2192.2	0.915	6918	142.4	48.6
The Netherlands	18.3	1122.5	0.955	16,639	49.0	339.8
Romania	18.9	353.8	0.845	-	60.5	-
Spain	47.9	1591.6	0.918	6953	79.9	87.0
Sweden	10.6	559.1	0.959	14,380	113.1	127.1
Turkey	87.7	1220.3	0.853	11,911	47.6	250.1
United Kingdom	69.5	3281.6	0.946	10,847	133.3	81.4

* GDP: Gross Domestic Product; HDI: Human Development Index; SBS ratio: surgery-by-surgeon ratio.

**Table 3:** Summary of work-related musculoskeletal disorders prevalence (%) reported in the 26 included studies.

Authors	Country	Sample Size	Specialty	WMSD Prevalence by Body Area									Overall WMSD Prevalence
				Neck	Upper Back	Lower Back	Shoulder	Elbow	Wrist	Hip	Knee	Ankle	
Amin et al., 2015 [63]	United Kingdom	82	Otorhinolaryngology	60.0%			45.0%		17.0%				74.0%
Babar-Craig et al., 2003 [64]	United Kingdom	325	Otorhinolaryngology	24.0%	29.%	19.0%							72.0%
Battevi et al., 2009 [55]	Italy	88	Gastroenterology				36.4%	17.0%	25.0%				40.0%
Boyle et al., 2022 [53]	Ireland	49	Otorhinolaryngology	59.2%	34.7%	32.6%	28.0%	10.0%	18.0%	1.5%	9.5%	7.5%	75.5%
Cass et al., 2014 [26]	United Kingdom	128	Gynecology	73.4%			80.5%		69.5%				99.0%
Dixon et al., 2023 [68]	Multiple (Europe)	259	Multiple	67.6%		73.7%	70.7%	26.6%	59.8%	17.0%	27.0%	20.1%	90.0%
Durden et al., 2023 [65]	United Kingdom	37	Multiple	37.8%	32.4%	43.2%	48.7%	5.4%	27.0%	16.2%	21.6%	8.1%	86.5%
Giagio et al., 2019 [33]	Italy	76	Multiple	78.9%	55.3%	71.1%	51.3%	3.9%	26.3%	14.5%	18.4%	18.4%	
Giberti et al., 2014 [56]	Italy	17	Multiple		29.4%	5.9%							41.2%
Gutierrez-Diez et al., 2017 [60]	Spain	129	Multiple	51.0%	44.0%	54.0%	29.0%	12.0%	28.0%				90.0%
Hyer et al., 2015 [66]	United Kingdom	518	Ophthalmology	31.8%	15.4%	41.7%							62.4%
Kaya et al., 2008 [62]	Turkey	82	Multiple	72.0%		70.0%							
Lakomek et al., 2025 [52]	Germany	751	Otorhinolaryngology	81.6%	55.1%	55.9%	54.5%			20.7%	22.4%	17.0%	82.0%
Lobo et al., 2019 [61]	Spain	407	Otorhinolaryngology	48.9%		60.2%	18.8%		16.3%				89.9%
Mal and Costello, 2002 [7]	United Kingdom	367	Otorhinolaryngology				24.0%						
Matern and Koneczny, 2007 [20]	Germany	425	Multiple	60.0%		85.0%	39.0%						84.0%
Morandeira-Rivas et al., 2012 [22]	Spain	78	Multiple	54.0%		53.0%	51.0%		49.0%		22.0%		81.0%
Quinn and Moohan, 2015 [54]	Ireland	53	Gynecology	41.5%	28.3%	28.3%	43.4%	15.1%	20.8%		37.7%	20.8%	
Rata et al., 2021 [18]	Romania	95	Multiple	55.8%	46.3%	74.7%	46.3%	17.7%	16.8%	11.6%	31.6%	4.2%	95.8%
Rata et al., 2021 [18]	Romania	6	General surgery	83.3%	33.3%	100.0%	33.3%	33.3%	0.0%	33.3%	66.7%	0.0%	
Rata et al., 2021 [18]	Romania	10	Vascular surgery	30.0%	80.0%	80.0%	40.0%	0.0%	10.0%	30.0%	10.0%	0.0%	
Rata et al., 2021 [18]	Romania	14	Plastic surgery	71.4%	71.4%	71.4%	14.3%	0.0%	28.6%	14.3%	28.6%	14.3%	
Rata et al., 2021 [18]	Romania	21	Neurosurgery	52.4%	47.6%	90.5%	38.1%	47.6%	14.3%	0.0%	23.8%	0.0%	
Rata et al., 2021 [18]	Romania	12	Orthopedic surgery	50.0%	50.0%	66.7%	66.7%	0.0%	33.3%	16.7%	33.3%	0.0%	
Rata et al., 2021 [18]	Romania	8	Urology	100.0%	50.0%	25.0%	50.0%	0.0%	25.0%	0.0%	50.0%	25.0%	
Rata et al., 2021 [18]	Romania	8	Cardiac surgery	50.0%	25.0%	75.0%	75.0%	0.0%	0.0%	0.0%	50.0%	0.0%	
Rata et al., 2021 [18]	Romania	8	Thoracic surgery	25.0%	25.0%	75.0%	25.0%	0.0%	0.0%	0.0%	0.0%	0.0%	
Rata et al., 2021 [18]	Romania	8	Gynecology	50.0%	0.0%	75.0%	100.0%	25.0%	25.0%	25.0%	50.0%	0.0%	
Rimmer et al., 2016 [69]	Multiple (Europe)	250	Otorhinolaryngology	60.3%		59.8%	50.8%		17.9%		10.6%		80.0%
Ruitenburg et al., 2012 [57]	The Netherlands	91	General surgery	35.0%	19.0%	24.0%	23.0%	4.0%	15.0%	4.0%	2.0%	2.0%	
Sari et al., 2010 [58]	The Netherlands	55	Multiple	15.0%		26.0%	45.0%						73.0%
Stomberg et al., 2010 [51]	Sweden	101	Gynecology	50.0%	24.0%	55.0%	51.0%	6.0%	14.0%		27.0%		70.0%
Stomberg et al., 2010 [51]	Sweden	103	General surgery	44.0%	21.0%	44.0%	38.0%	7.0%	26.0%		26.0%		70.0%
Tjiam et al., 2014 [59]	The Netherlands	285	Urology	59.3%			51.2%	26.0%	21.4%				86.0%
Vijendren et al., 2016 [67]	United Kingdom	323	Otorhinolaryngology	29.7%					9.0%				47.4%

WMSD: work-related musculoskeletal disorders.

**Table 4:** Meta-analysis results of work-related musculoskeletal disorders prevalence (overall and by body area) according to surgeons’ age.

Body Area	Age < 45 Years						Age > 45 Years						Evolution Profile
	N	S. Size	I^2^	ES	95% CI	Model	N	S. Size	I^2^	ES	95% CI	Model	
Neck	8	680	85.56	46.0%	(33.0–58.9)	Random	7	2181	96.07	53.0%	(36.6–69.4)	Random	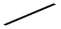
Upper back	6	547	83.20	34.7%	(23.0–46.4)	Random	5	1424	97.40	31.3%	(10.1–52.5)	Random	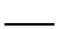
Lower back	8	680	85.76	46.1%	(33.0–59.1)	Random	6	1831	93.38	43.9%	(30.4–57.5)	Random	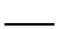
Shoulder	9	768	59.33	38.9%	(32.0–45.8)	Random	7	2030	95.97	41.3%	(27.4–55.1)	Random	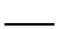
Elbow	7	635	69.62	9.8%	(5.6–14.0)	Random	3	423	93.45	12.5%	(0.0–26.0)	Random	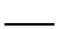
Wrist	8	713	62.96	24.6%	(18.6–30.5)	Random	5	912	11.26	17.6%	(14.9–20.4)	Fixed	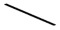
Hip	3	262	70.18	9.3%	(2.6–16.0)	Random	2	788	0.0	20.4%	(0.0–23.6)	Fixed	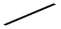
Knee	6	496	92.83	22.2%	(9.1–35.3)	Random	3	889	0.0	22.8%	(19.7–25.9)	Fixed	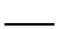
Ankle	4	315	82.91	9.2%	(2.2–16.1)	Random	2	788	69.50	13.7%	(5.2–22.1)	Random	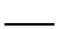
Overall	6	548	85.01	74.4%	(56.0–92.9)	Random	8	2198	80.89	75.9%	(66.2–85.6)	Random	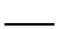

N: number of studies. S. size: sample size. ES: effect sizes. 95% CI: 95% confidence interval.

**Table 5:** Meta-analysis results of work-related musculoskeletal disorders prevalence (overall and by body area) according to surgeons’ years of experience.

Body Area	Experience < 10 Years						Experience > 10 Years						Evolution Profile
	N	S. Size	I^2^	ES	95% CI	Model	N	S. Size	I^2^	ES	95% CI	Model	
Neck	3	211	86.16	32.9%	(12.5–53.4)	Random	9	2249	94.48	56.8%	(43.2–70.5)	Random	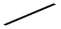
Upper back	3	173	0.0	23.5%	(16.3–30.7)	Fixed	5	1152	87.63	44.7%	(31.6–57.7)	Random	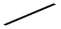
Lower back	4	228	84.36	25.9%	(9.5–42.3)	Random	6	1559	26.80	58.3%	(54.5–62.0)	Fixed	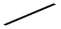
Shoulder	3	211	0.0	40.9%	(32.3–49.6)	Fixed	9	2014	94.19	42.3%	(30.2–54.4)	Random	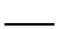
Elbow	2	156	46.22	8.6%	(4.0–13.2)	Fixed	6	774	88.38	13.5%	(6.3–20.7)	Random	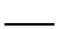
Wrist	2	156	0.0	24.0%	(16.3–31.6)	Fixed	9	1586	76.51	18.4%	(13.9–22.9)	Random	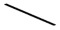
Hip	-	-	-	-	-		3	922	69.20	16.3%	(10.0–22.6)	Random	-
Knee	2	156	29.60	29.1%	(20.6–37.5)	Fixed	4	1023	21.38	23.0%	(20.1–26.0)	Fixed	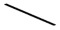
Ankle	-	-	-	-	-		3	922	92.24	12.8%	(3.0–22.7)	Random	-
Overall	3	175	36.29	66.4%	(54.3–78.4)	Fixed	9	2261	92.31	74.7%	(61.2–88.1)	Random	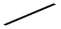

N: number of studies. S. size: sample size. ES: effect sizes. 95% CI: 95% confidence interval.

**Table 6:** Meta-analysis results of work-related musculoskeletal disorders prevalence (overall and by body area) according to the Surgery-By-Surgeon ratio.

Body Area	SBS Ratio < 125						SBS Ratio > 125						Evolution Profile
	N	S. Size	I^2^	ES	95% CI	Model	N	S. Size	I^2^	ES	95% CI	Model	
Neck	12	2205	89.58	47.5%	(38.7–56.3)	Random	8	1893	95.04	52.1%	(36.1–68.1)	Random	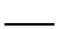
Upper back	8	1204	87.06	32.8%	(22.8–42.7)	Random	4	1046	96.23	30.0%	(9.6–50.4)	Random	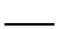
Lower back	10	1689	93.82	40.4%	(27.8–52.9)	Random	7	1608	94.26	51.4%	(34.9–67.8)	Random	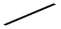
Shoulder	11	1494	89.34	40.1%	(30.6–49.6)	Random	7	1811	84.88	43.1%	(34.4–51.8)	Random	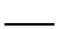
Elbow	6	432	56.44	9.8%	(5.3–14.2)	Random	4	580	92.40	10.6%	(1.7–19.5)	Random	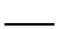
Wrist	11	1450	90.21	26.6%	(18.7–34.4)	Random	4	580	45.28	19.0%	(15.4–22.5)	Fixed	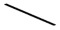
Hip	3	162	82.17	9.7%	(0.0–20.5)	Random	2	842	97.44	12.4%	(0.0–28.8)	Random	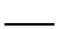
Knee	5	293	59.83	20.2%	(12.1–28.3)	Random	4	1046	96.94	18.9%	(4.9–33.0)	Random	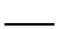
Ankle	4	215	47.20	12.1%	(7.4–16.7)	Fixed	2	842	98.02	9.5%	(0.0–24.2)	Random	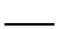
Overall	12	2181	88.65	71.4%	(60.2–82.6)	Random	6	1720	7.07	81.1%	(76.9–85.4)	Fixed	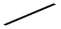

N: number of studies; S. size: sample size; ES: effect sizes; 95% CI: 95% confidence interval; SBS ratio: Surgery-By-Surgeon ratio.

**Table 7:** Meta-regression results of relationships between gross domestic product, human development index, case load (hour per week), year of publication and work-related musculoskeletal disorders prevalence.

Body Area	GDP		HDI		Case Load (Hour per Week)		Year of Publication	
	Pearson’s r	*p*-Value	Pearson’s r	*p*-Value	Pearson’s r	*p*-Value	Pearson’s r	*p*-Value
Neck	−0.007	0.971	−0.240	0.202	0.257	0.539	0.392	0.064
Upper back	−0.049	0.829	−0.326	0.139	0.530	0.358	0.624	**0.023**
Lower back	−0.219	0.272	−0.603	**<0.001**	0.587	0.221	0.228	0.333
Shoulder	0.024	0.902	−0.148	0.452	−0.286	0.583	0.281	0.218
Elbow	−0.084	0.724	−0.061	0.797	−0.524	0.476	0.169	0.600
Wrist	0.393	0.052	0.267	0.196	0.288	0.638	0.073	0.774
Hip	0.190	0.499	−0.107	0.704	−	−	0.618	0.139
Knee	−0.248	0.305	−0.380	0.109	−0.983	0.118	0.034	0.917
Ankle	0.370	0.159	0.421	0.104	−	−	0.212	0.614
Overall	−0.074	0.764	−0.096	0.696	0.540	0.269	0.469	**0.043**

GDP: Gross Domestic Product; HDI: Human Development Index. Values in bold indicate significant correlations.

**Table 8:** Sensitivity analysis: effect of using the Nordic Musculoskeletal disorders Questionnaire on the pooled prevalence for each body area.

Body Area	Studies with NMQ						Studies Without NMQ						Absolute Difference
	N	S. Size	I^2^	ES	95% CI	Model	N	S. Size	I^2^	ES	95% CI	Model	
Neck	4	337	65.92	55.5%	(41.4–69.5)	Random	19	4365	94.97	50.5%	(41.3–59.8)	Random	5.0%
Upper back	5	354	13.61	43.8%	(36.9–50.7)	Fixed	8	1991	95.6	28.3%	(16.3–40.2)	Random	15.5%
Lower back	5	354	93.72	49.4%	(21.6–77.2)	Random	15	3547	95.46	48.5%	(37.6–59.4)	Random	0.9%
Shoulder	4	337	64.62	42.2%	(30.0–54.5)	Random	17	3572	93.62	43.8%	(35.3–52.2)	Random	1.6%
Elbow	4	337	71.45	9.3%	(3.3–15.2)	Random	8	1029	89.74	13.8%	(7.0–20.6)	Random	4.5%
Wrist	4	337	22.14	23.2%	(18.1–28.3)	Fixed	14	2297	92.67	25.9%	(19.0–32.9)	Random	2.7%
Hip	3	208	0	13.2%	(8.3–18.2)	Fixed	4	1150	96.26	10.8%	(0.8–20.8)	Random	2.4%
Knee	3	208	35.66	23.5%	(16.9–30.1)	Fixed	8	1485	94.56	21.0%	(11.6–30.4)	Random	2.5%
Ankle	3	208	72.05	9.4%	(1.2–17.6)	Random	5	1203	94	13.1%	(4.5–21.7)	Random	3.7%
Overall	4	278	68.22	80.9%	(60.4–101.4)	Random	17	4227	87.64	74.9%	(67.1–82.7)	Random	6.0%

N: number of studies; S. size: sample size; ES: effect sizes; 95% CI: 95% confidence interval; NMQ: Nordic Musculoskeletal disorders Questionnaire.

## Data Availability

Data available on request.

## Supplementary Materials

The following supporting information can be downloaded at: https://www.mdpi.com/article/10.3390/ijerph23030398/s1, Table S1: PRISMA 2020 Checklist.

## Abbreviations

The following abbreviations are used in this manuscript:

AXIS	Tool for evaluating cross-sectional studies
BMI	Body mass index
GDP	Gross Domestic Product
HDI	Human Development Index
NMQ	Nordic Musculoskeletal Disorders Questionnaire
PECO	Population, Exposure, Comparator, and Outcomes guidelines
PRISMA	Preferred Reporting Items for Systematic reviews and Meta-Analyses
SBS	Surgery by Surgeon
WMSDs	Work-related musculoskeletal disorders

## Appendix A. AXIS

**Table A1:** Quality appraisal of each included study according to AXIS tool.

	1. Were the Aims/Objectives of the Study Clear?	2. Was the Study Design Appropriate for the Stated Aim(s)?	3. Was the Sample Size Justified?	4. Was the Target/Reference Population Clearly Defined? (Is it Clear Who the Research Was About?)	5. Was the Sample Frame Taken from an appropriate Population Base so That It Closely Represented the Target/Reference Population Under Investigation?	6. Was the Selection Process Likely to Select Subjects/Participants that Were Representative of the Target/Reference Population Under Investigation?	7. Were Measures Undertaken to Address and Categorize Non-Responders?	8. Were the Risk Factor and Outcome Variables Measured Appropriate to the Aims of the Study?	9. Were the Risk Factor and Outcome Variables Measured Correctly Using Instruments/Measurements that Had Been Trialed, Piloted or Published Previously?	10. Is it Clear What Was Used to Determined Statistical Significance and/or Precision Estimates? (e.g., *p* Values, cis)	11. Were the Methods (Including Statistical Methods) Sufficiently Described to Enable Them to Be Repeated?	12. Were the Basic Data Adequately Described?	13. Does the Response Rate Raise Concerns About Non-Response Bias?	14. If Appropriate, Was information About Non-Responders Described?	15. Were the Results Internally Consistent?	16. Were the Results for the Analyses Described in the Methods, Presented?	17. Were the Authors’ Discussions and Conclusions Justified by the Results?	18. Were the Limitations of the Study Discussed?	19. Were There Any Funding Sources or Conflicts of Interest That May Affect the Authors’ Interpretation of The Results? *	20. Was Ethical Approval or Consent of Participants Attained?
Amin et al., 2015 [63]	Yes	Yes	No	Yes	Yes	Yes	No	Yes	Yes	Yes	Yes	Yes	No	NA	Yes	Yes	Yes	Yes	No	No
Babar-Craig et al., 2003 [64]	Yes	Yes	No	Yes	Yes	Yes	No	Yes	Yes	No	Yes	Yes	No	NA	Yes	Yes	Yes	No	No	Yes
Battevi et al., 2009 [55]	Yes	Yes	No	Yes	Yes	Yes	No	Yes	Yes	No	Yes	Yes	No	NA	Yes	Yes	Yes	No	No	Yes
Boyle et al., 2022 [53]	Yes	Yes	No	Yes	Yes	Yes	No	Yes	Yes	Yes	Yes	Yes	No	NA	Yes	Yes	Yes	Yes	No	Yes
Cass et al., 2014 [26]	Yes	Yes	No	Yes	Yes	Yes	No	Yes	Yes	Yes	Yes	Yes	No	NA	Yes	Yes	Yes	Yes	No	Yes
Dixon et al., 2023 [68]	Yes	Yes	No	Yes	Yes	Yes	No	Yes	Yes	No	Yes	Yes	No	NA	Yes	Yes	Yes	Yes	No	No
Durden et al., 2023 [65]	Yes	Yes	No	Yes	Yes	Yes	No	Yes	Yes	Yes	Yes	Yes	No	NA	Yes	Yes	Yes	Yes	No	No
Giagio et al., 2019 [33]	Yes	Yes	Yes	Yes	Yes	Yes	No	Yes	Yes	Yes	Yes	Yes	No	NA	Yes	Yes	Yes	Yes	No	Yes
Giberti et al., 2014 [56]	Yes	Yes	No	Yes	Yes	Yes	No	Yes	Yes	Yes	Yes	Yes	No	NA	Yes	Yes	Yes	Yes	No	Yes
Gutierrez-Diez et al., 2017 [60]	Yes	Yes	No	Yes	Yes	Yes	No	Yes	Yes	Yes	Yes	Yes	No	NA	Yes	Yes	Yes	No	No	Yes
Hyer et al., 2015 [66]	Yes	Yes	No	Yes	Yes	Yes	No	Yes	Yes	Yes	Yes	Yes	No	NA	Yes	Yes	Yes	Yes	No	Yes
Kaya et al., 2008 [62]	Yes	Yes	No	Yes	Yes	Yes	No	Yes	Yes	No	Yes	Yes	No	NA	Yes	Yes	Yes	No	No	Yes
Lakomek et al., 2025 [52]	Yes	Yes	No	Yes	Yes	Yes	No	Yes	Yes	No	Yes	Yes	No	NA	Yes	Yes	Yes	Yes	No	Yes
Lobo et al., 2019 [61]	Yes	Yes	No	Yes	Yes	Yes	No	Yes	Yes	Yes	Yes	Yes	No	NA	Yes	Yes	Yes	Yes	No	Yes
Mal and Costello, 2002 [7]	Yes	Yes	No	Yes	Yes	Yes	No	Yes	Yes	Yes	Yes	Yes	No	NA	Yes	Yes	Yes	No	No	Yes
Matern and Koneczny, 2007 [20]	Yes	Yes	No	Yes	Yes	Yes	No	Yes	Yes	No	Yes	Yes	No	NA	Yes	Yes	Yes	No	No	Yes
Morandeira-Rivas et al., 2012 [22]	Yes	Yes	No	Yes	Yes	Yes	No	Yes	Yes	Yes	Yes	Yes	No	NA	Yes	Yes	Yes	Yes	No	Yes
Quinn and Moohan, 2015 [54]	Yes	Yes	No	Yes	Yes	Yes	No	Yes	Yes	Yes	Yes	Yes	No	NA	Yes	Yes	Yes	No	No	Yes
Rata et al., 2021 [18]	Yes	Yes	No	Yes	Yes	Yes	No	Yes	Yes	Yes	Yes	Yes	No	NA	Yes	Yes	Yes	Yes	No	Yes
Rimmer et al., 2016 [69]	Yes	Yes	No	Yes	Yes	Yes	No	Yes	Yes	Yes	Yes	Yes	No	NA	Yes	Yes	Yes	No	No	No
Ruitenburg et al., 2012 [57]	Yes	Yes	No	Yes	Yes	Yes	No	Yes	Yes	Yes	Yes	Yes	No	NA	Yes	Yes	Yes	No	No	Yes
Sari et al., 2010 [58]	Yes	Yes	No	Yes	Yes	Yes	No	Yes	Yes	Yes	Yes	Yes	No	NA	Yes	Yes	Yes	Yes	No	Yes
Stomberg et al., 2010 [51]	Yes	Yes	No	Yes	Yes	Yes	No	Yes	Yes	Yes	Yes	Yes	No	NA	Yes	Yes	Yes	Yes	No	Yes
Tjiam et al., 2014 [59]	Yes	Yes	No	Yes	Yes	Yes	No	Yes	Yes	Yes	Yes	Yes	No	NA	Yes	Yes	Yes	Yes	No	Yes
Vijendren et al., 2016 [67]	Yes	Yes	No	Yes	Yes	Yes	No	Yes	Yes	No	Yes	Yes	No	NA	Yes	Yes	Yes	Yes	No	Yes

*: An absence of conflict (No answer) is considered a mark of quality and is therefore counted as a “Yes” in the computation of the quality score.

## Appendix B

**Table A2:** Quality of evidence description based on the Grading of Recommendations, Assessment, Development and Evaluation system (GRADE).

Body Area	Number of Studies	Certainty Assessment						Effect			Overall Quality
		Study Design	Publication Bias (Egger Test)	Indirectness	Inconsistency	Imprecision	Risk of Bias	n	Event Rate	(95% CI)	
Neck	23	Observational	Not serious (*p* = 0.145)	Not serious	Serious (I^2^ = 91.81%)	Not serious	Not serious	4797	51.3%	(43.7–58.9)	Very low ⬤◯◯◯
Upper back	13	Observational	Serious (*p* = 0.006)	Not serious	Serious (I^2^ = 96.02%)	Serious	Not serious	2440	34.2%	(24.2–44.1)	Very low ⬤◯◯◯
Lower back	20	Observational	Not serious (*p* = 0.173)	Not serious	Serious (I^2^ = 92.78%)	Not serious	Not serious	3996	52.1%	(43.1–61.0)	Very low ⬤◯◯◯
Shoulder	21	Observational	Not serious (*p* = 0.067)	Not serious	Serious (I^2^ = 89.33%)	Not serious	Not serious	4004	43.1%	(36.4–49.8)	Very low ⬤◯◯◯
Elbow	12	Observational	Serious (*p* < 0.001)	Not serious	Serious (I^2^ = 90.47%)	Serious	Not serious	1461	7.8%	(5.0–10.6)	Very low ⬤◯◯◯
Wrist	18	Observational	Serious (*p* < 0.001)	Not serious	Serious (I^2^ = 94.67%)	Not serious	Not serious	2729	21.3%	(16.1–26.5)	Very low ⬤◯◯◯
Hip	7	Observational	Serious (*p* = 0.030)	Not serious	Serious (I^2^ = 92.73%)	Serious	Not serious	1453	8.1%	(4.1–12.0)	Very low ⬤◯◯◯
Knee	12	Observational	Serious (*p* = 0.003)	Not serious	Serious (I^2^ = 92.23%)	Serious	Not serious	2038	20.8%	(14.7–27.0)	Very low ⬤◯◯◯
Ankle	8	Observational	Serious (*p* = 0.010)	Not serious	Serious (I^2^ = 91.98%)	Serious	Not serious	1506	6.1%	(3.1–9.0)	Very low ⬤◯◯◯
Overall	21	Observational	Not serious (*p* = 0.506)	Not serious	Serious (I^2^ = 86.03%)	Not serious	Not serious	4505	75.8%	(68.6–83.1)	Very low ⬤◯◯◯

Overall quality significance: ⬤◯◯◯ = Very low; ⬤⬤◯◯ = Low; ⬤⬤⬤◯ = Moderate; ⬤⬤⬤⬤ = High.
